# Can Preoperative Hounsfield Unit Measurement Help Predict Mechanical Failure in Metastatic Spinal Tumor Surgery?

**DOI:** 10.3390/jcm13237017

**Published:** 2024-11-21

**Authors:** Hyung Rae Lee, Jae Hwan Cho, Sang Yun Seok, San Kim, Dae Wi Cho, Jae Hyuk Yang

**Affiliations:** 1 Department of Orthopedic Surgery, Korea University Medical Center, Anam Hospital, Seoul 02841, Republic of Korea; drhrleeos@gmail.com (H.R.L.); kuspine@korea.ac.kr (J.H.Y.); 2 Department of Orthopedic Surgery, Asan Medical Center, University of Ulsan College of Medicine, Seoul 05505, Republic of Korea; kimrlatks2@naver.com (S.K.); zozo5313@gmail.com (D.W.C.); 3 Department of Orthopedic Surgery, Daejeon Eulji Medical Center, University of Eulji College of Medicine, Daejeon 35233, Republic of Korea; oper251@hanmail.net

**Keywords:** metastatic spinal tumors, instrumentation without fusion, Hounsfield units, mechanical failure, cement-augmented screws, propensity score matching

## Abstract

**Background/Objectives:** This study aimed to identify risk factors associated with mechanical failure in patients undergoing spinal instrumentation without fusion for metastatic spinal tumors. **Methods:** We retrospectively evaluated data from 220 patients with spinal tumors who underwent instrumentation without fusion. Propensity scores were used to match preoperative variables, resulting in the inclusion of 24 patients in the failure group (F group) and 72 in the non-failure group (non-F group). Demographic, surgical, and radiological characteristics were compared between the two groups. Logistic regression and Kaplan–Meier survival analyses were conducted to identify predictors of mechanical failure. **Results:** Propensity score matching resulted in a balanced distribution of covariates. Lower Hounsfield unit (HU) values at the lowest instrumented vertebra (LIV) were the only independent predictor of implant failure (*p* = 0.037). A cutoff value of 127.273 HUs was determined to predict mechanical failure, with a sensitivity of 59.1%, specificity of 73.4%, and area under the curve of 0.655 (95% confidence interval: 0.49–0.79). A significant difference in survival was observed between the groups with HU values above and below the cutoff (*p* = 0.0057). Cement-augmented screws were underutilized, with an average of only 0.2 screws per patient in the F group. **Conclusions:** Preoperative LIV HU values < 127.273 were strongly associated with an increased risk of mechanical failure following spinal instrumentation without fusion. Alternative surgical strategies including the use of cement-augmented screws are recommended for patients with low HU values.

## 1. Introduction

Spinal metastatic tumors arise from various primary cancers and often cause pain, neurological symptoms, and spinal instability [1,2,3]. Surgical intervention is essential, particularly in cases of cord compression that promote neurological deficits or instability caused by metastasis [1,4]. Common surgical approaches include decompression or corpectomy with fixation, as well as en bloc excision and fixation. In many cases, fusion is not performed, and fixation alone is often preferred [1,4,5,6].

Achieving stable arthrodesis in patients with spinal tumors is a significant challenge. Although fusion is typically performed following spinal instrumentation to enhance stability, the environment in metastatic spine conditions is often suboptimal for fusion [1,3,5,7,8,9]. Factors such as poor bone quality, the impact of adjuvant therapies, and the typically short survival time of these patients complicate the fusion process [2,3]. Consequently, many clinicians opt for instrumentation alone without performing additional fusion after decompression, to provide immediate stability without the burden of achieving long-term bone fusion. Yee et al. reported that the fusion rate in patients with spinal metastatic tumors was only 28% [6], underscoring the difficulty of achieving successful arthrodesis in this population.

Although this approach can mitigate some immediate surgical challenges, it is associated with risks. The overall implant failure rate for these procedures ranges from approximately 3% to 13.8% [1,5]. Despite previous investigations of implant failure rates in patients with spinal metastatic tumors, the specific risk factors contributing to mechanical failure remain largely unknown. Considering the extended survival times of some patients owing to advances in cancer treatment, identifying these risk factors has become increasingly important [10,11,12].

This study aimed to determine the incidence of mechanical failure following decompression and fixation surgery in patients with metastatic spinal tumors. Additionally, we identified and analyzed the risk factors associated with such mechanical failures. Gaining this understanding is crucial for improving surgical outcomes and optimizing the management of spinal metastases in this challenging patient population.

## 2. Materials and Methods

### 2.1. Study Design and Patients

This study was approved by the institutional review board (IRB number: S2023-0763-0001, approved on 29 March 2023). The requirement for informed consent was waived owing to the retrospective nature of the study. This study was designed and reported in accordance with the Strengthening the Reporting of Observational Studies in Epidemiology (STROBE) statement for cohort studies. Between 2014 and 2020, 220 patients with spinal tumors who underwent decompression and instrumentation surgery, without fusion, for metastatic spinal tumors were enrolled in this study. Two experienced spine surgeons (JHC and JWP) performed all surgeries at a single institution. The primary indications for surgery included severe pain or neurological deterioration resulting from pathologic fractures or metastatic spinal cord compression [10,13].

The exclusion criteria were as follows: use of a fusion substrate during surgery; incomplete medical records, clinical scores, or radiographs; loss to follow-up or death within 6 months postoperatively; and surgery without instrumentation, including procedures such as cementing or decompression alone. After applying these exclusion criteria, the remaining patients were subjected to propensity score matching (PSM) based on preoperative variables such as age, sex, and bone mineral density (BMD) [14]. PSM was implemented to minimize selection bias and ensure comparability between the failure (F) and non-failure (non-F) groups. This matching resulted in a cohort with a 1:3 ratio that included 24 and 72 patients in the F and non-F groups, respectively. Thus, well-aligned groups were created for subsequent analyses.

### 2.2. Variables

A comprehensive set of variables was analyzed to assess the factors related to mechanical failure after instrumentation without fusion in patients with spinal tumors. These variables included demographic factors, tumor-related factors, and radiological assessments.

### 2.3. Demographic Factors

We collected data on age, sex, height, body mass index (BMI), and BMD from all patients enrolled. Additionally, we documented the type of surgery performed, including the number of laminectomy levels, fixation levels, screws placed in the tumor, the number of rods, and the use of cemented screws. We also tracked the time to mechanical failure, incidence of symptomatic local recurrence, and rate of reoperation to provide insights into the durability of surgical interventions.

### 2.4. Tumor-Related Factors

The origin of the tumor (e.g., lung, breast, or kidney) and the extent of tumor involvement, which included the number of vertebral levels affected by metastasis and whether the tumor had spread from the spinal column to other regions, were recorded. The number of vertebral levels instrumented above and below the affected area was also documented, typically involving two levels above and below the tumor-affected vertebrae.

### 2.5. Radiological Assessment

**Figure 1:**
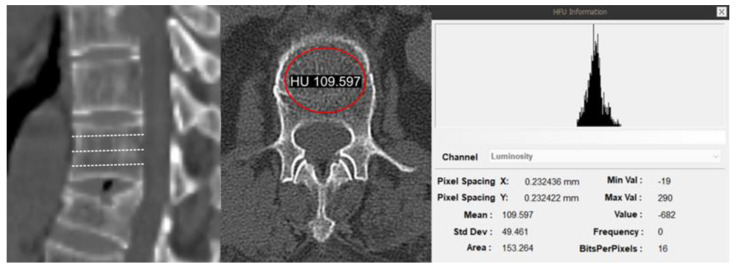
Preoperative computed tomography scan illustrating the method for measuring Hounsfield units (HUs) at the vertebral body. HU measurements were obtained from the trabecular bone at the upper and lowest instrumented vertebrae, represented by dashed lines indicating three axial slices per vertebra for accurate assessment.

Radiological assessment was a critical component of this study. Hounsfield units (HUs) were measured on preoperative computed tomography (CT) scans to evaluate bone quality [15,16]. HU measurements were taken at specific vertebral levels, including the upper instrumented vertebra (UIV) and lowest instrumented vertebra (LIV). HU values were obtained by averaging the measurements across three axial slices of the vertebral body (VB), avoiding the cortical bone and focusing on the trabecular bone (Figure 1) [17]. Additional radiological factors such as Bilsky grades, spinal instability neoplastic scores (SINSs), and the extent of VB collapse were also assessed [12,18].

### 2.6. Mechanical Failures

Mechanical failure of the instrumentation was defined based on clinical experience at our institution and established research criteria. Instrumentation failure was characterized by screw loosening, rod fracture or displacement, and subsidence of the cage, cement, or bone [19].

### 2.7. Statistical Analysis

Statistical analyses were conducted to identify the factors associated with mechanical failure. Logistic regression analysis was used to identify independent predictors of mechanical failure due to its suitability for binary outcome variables. Receiver operating characteristic (ROC) curve analysis was employed to determine the optimal cutoff for Hounsfield unit values, as this method provides sensitivity and specificity estimates. A Kaplan–Meier survival analysis was conducted to compare time-to-event outcomes between groups, providing insights into implant survival over time. In addition to propensity score matching, multivariate analyses included adjustments for confounding factors such as age, body mass index, and preoperative radiotherapy to further enhance the robustness of our findings. All statistical analyses were performed using IBM SPSS Statistics version 21.0 for Windows (IBM Corp., Armonk, NY, USA). Statistical significance was set at *p* < 0.05.

## 3. Results

### 3.1. Demographic Characteristics

The PSM resulted in a balanced distribution of covariates between the F and non-F groups, comprising 24 and 72 patients, respectively, achieving a 1:3 ratio. Specifically, the mean age difference between the groups was reduced from 4.22 years pre-matching to 0.61 years post-matching. No significant differences in age, sex, BMI, BMD, number of vertebral bodies involved, mode of surgery, PreopRT, major organ involvement, number of other bone lesions, or postoperative RT were observed between the two groups. The F group had a longer mean follow-up period than the non-F group (21.6 ± 18.8 vs. 15.4 ± 15.8 months), although this difference was not significant (*p* = 0.121, Table 1). The demographic and clinical characteristics of the 24 patients in the F group are shown in Table 2. The table outlines key factors, such as tumor origin, location, mode of surgery, number of laminectomies, fixation levels, screws placed in the tumor, and the type and timing of implant failures. The time to failure among the patients ranged from 0.25 to 27 months. Implant failure resulted from screw loosening (16 cases), rod breakage (2 cases), and cage subsidence. A significant proportion of patients (79.2%) in the F group required reoperation because of symptomatic implant failure.

### 3.2. Radiological Characteristics

Radiological characteristics, including the Bilsky grade, SINS, and VB collapse, were compared between the two groups (Table 3). No significant differences in the Bilsky grade, specific components of the SINS, or HU measurements at the UIV were identified between the two groups. However, the HU measurements at the LIV were significantly lower in the F group than in the non-F group (142.2 ± 62.2 vs. 178.9 ± 84.5, *p* = 0.042).

### 3.3. Surgical Characteristics

The surgical characteristics of the two groups are shown in Table 4. Although there were no significant differences in the levels of laminectomy, fixation, or the number of screws used between the two groups, the F group experienced a higher rate of symptomatic local recurrence (50.0% vs. 27.8%, *p* = 0.08) and a significantly higher rate of reoperation (79.2% vs. 33.3%, *p* < 0.001) than the non-F group.

### 3.4. Logistic Regression and ROC Analyses

**Figure 2:**
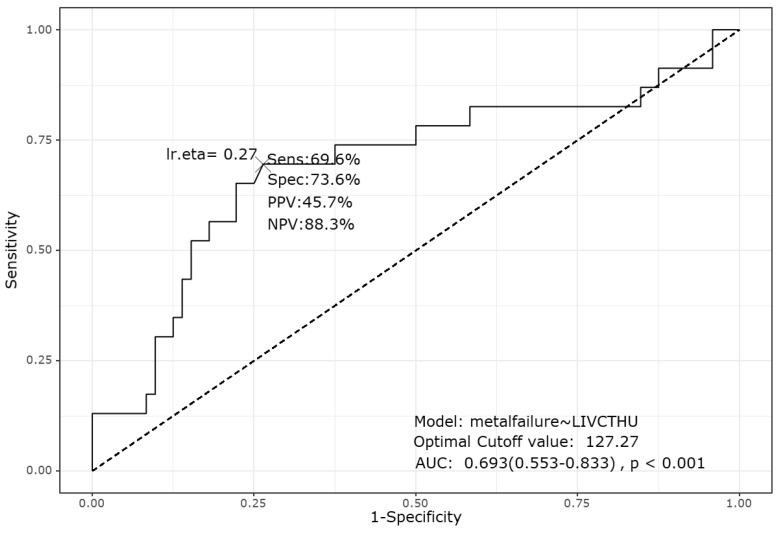
Receiver operating characteristic curve analysis for Hounsfield units (HUs) at the lowest instrumented vertebra (LIV), identifying a cutoff value of 127.273 for predicting mechanical failure after instrumentation without fusion. The analysis showed a sensitivity of 69.6% and a specificity of 73.6%, with an area under the curve (AUC) of 0.693 (95% confidence interval: 0.55–0.83) and *p* < 0.001. PPV, positive predictive value; NPV, negative predictive value.

**Figure 3:**
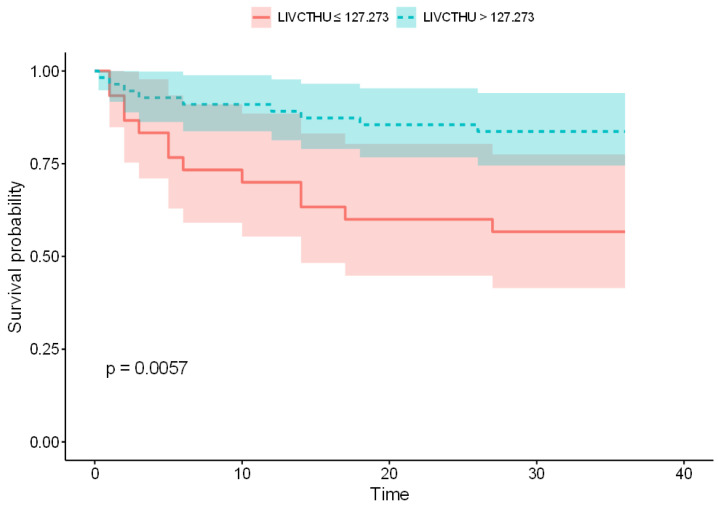
Kaplan–Meier survival curve comparing the time to mechanical failure between patients with Hounsfield unit (HU) values above and below the identified cutoff at the lowest instrumented vertebra (LIV). The survival curve demonstrates a significant difference in implant survival, with *p* = 0.0057, indicating that patients with LIV HU values < 127.273 have a higher risk of earlier mechanical failure.

The logistic regression analysis revealed that lower LIV HU values and Bilsky grades were associated with an increased risk of mechanical failure (Table 5). Although other factors such as PreopRT and the SINS showed trends toward significance, they did not reach statistical significance in this study. However, the multivariate analysis identified lower LIV HU values as the only independent predictor of postoperative implant failure (*p* = 0.037). Furthermore, the ROC analysis (Figure 2) established a CT-measured LIV HU value < 127.27 as the cutoff value for predicting implant failure. This cutoff value had a sensitivity, specificity, and area under the curve of 69.6%, 73.6%, and 0.693, respectively (95% confidence interval: 0.55–0.83, *p* < 0.01). This finding was further confirmed by Kaplan–Meier survival analysis, indicating a significant difference in implant survival between the groups with LIV HU values above and below the cutoff (*p* = 0.0057, Figure 3).

### 3.5. Case Analysis

#### 3.5.1. Case 1

**Figure 4:**
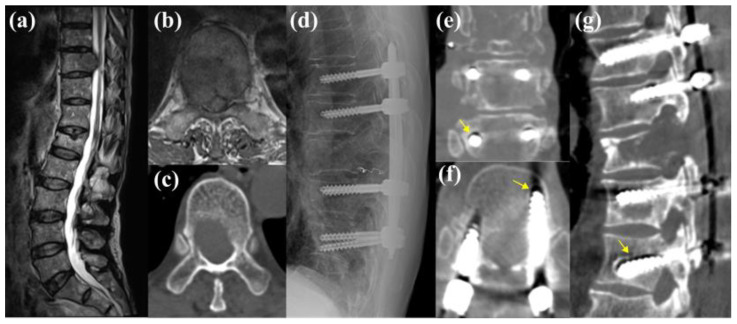
Representative case of a 70-year-old male patient with liver hepatocellular carcinoma metastasis to the T10 vertebra. (**a**–**c**) Preoperative magnetic resonance imaging and computed tomography (CT) imaging revealed the metastatic lesion, with a preoperative lowest instrumented vertebra Hounsfield unit value of 94.15, which is below the cutoff value associated with an increased risk of implant failure. (**d**) The patient underwent decompression with a partial corpectomy and posterior pedicle screw fixation from the T8 to T12. Postoperative plain radiographs initially showed an adequate hardware placement. (**e**–**g**) However, 14 months postoperatively, the patient experienced aggravated back pain, and subsequent CT imaging revealed loosening of the T12 pedicle screws (yellow arrows).

A representative case from the F group was a 70-year-old male patient with liver cancer that had metastasized to the T10 vertebra (Figure 4). Preoperative CT imaging revealed an HU value of 94.15 at the LIV (T12) and 151.852 at the UIV. These values, particularly the LIV HUs, were below the identified cutoff value of 127.27, indicating an increased risk of implant failure. The patient underwent decompression with a partial corpectomy and percutaneous pedicle screw fixation from T8 to T12. Approximately 14 months postoperatively, the patient’s back pain worsened, and CT tomography revealed a loosening of the T12 screws.

#### 3.5.2. Case 2

**Figure 5:**
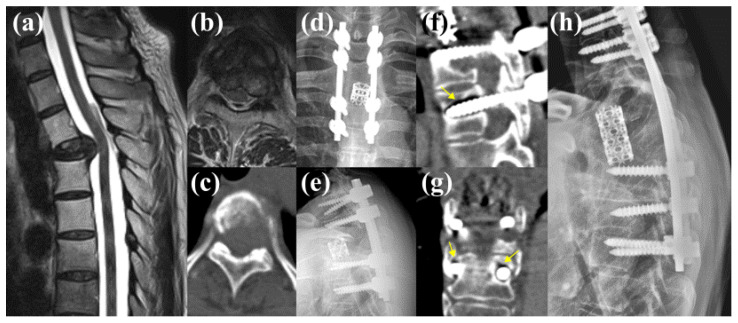
Case involving a 48-year-old female patient with renal cell carcinoma metastasis to the T4 vertebra. (**a**–**c**) Preoperative magnetic resonance imaging and computed tomography (CT) imaging revealed the metastatic lesion. (**d**,**e**) The patient underwent a T4 spondylectomy with mesh cage insertion and posterior fixation from T2 to T6. Preoperative computed tomography (CT) imaging showed an average Hounsfield unit value of 264.862 at the upper instrumented vertebra and 103.201 at the lowest instrumented vertebra. (**f**,**g**) Approximately 17 months postoperatively, the patient experienced aggravated back pain, and CT imaging revealed loosening of the bilateral pedicle screws at the T6 vertebra (yellow arrows), (**h**) leading to revision surgery extending the fixation from C7 to T7.

Another failed case involved a 48-year-old female patient with renal cell carcinoma metastasis to the T4 vertebra (Figure 5). The patient underwent a T4 spondylectomy with mesh cage insertion and posterior fixation from T2 to T6. Preoperative CT imaging showed an average HU value of 264.862 at the UIV and 103.201 at the LIV, with the LIV HU below the cutoff value of 127.27. Approximately 17 months postoperatively, the patient experienced aggravated back pain, and CT tomography revealed loosening of the bilateral pedicle screws at the T6 vertebra. Consequently, revision surgery was performed, extending the fixation from C7 to T7.

## 4. Discussion

This study aimed to identify risk factors associated with mechanical failure in patients undergoing instrumentation without fusion for metastatic spinal tumors. Among the various factors analyzed, lower HU values at the LIV were identified as a key predictor of implant failure. This result was consistent across multiple analyses, including logistic regression and Kaplan–Meier survival analyses, underscoring the critical role of bone quality, as measured by HUs, in the success of instrumentation without fusion in patients with metastatic spinal tumors.

The decision to perform instrumentation without fusion in these patients was influenced by several factors [1,6,10,11,20,21]. First, the average life expectancy of patients with spinal metastases is often limited to a few months, indicating that even with attempting fusion, patients frequently succumb to their illness before achieving bone union [4,7,22]. Second, the bone quality in these patients, particularly in the fusion bed, is often poor, thus further reducing the likelihood of successful fusion [5,6,12,23]. Previous studies have reported mixed outcomes for fixation alone [24]. Some studies have suggested that even in cases of rod fracture, patients may not always experience significant discomfort, implying that fixation alone might suffice [24]. However, other studies have indicated that additional stabilization efforts may be necessary to avoid complications. As patients’ survival rates improve, concerns regarding the impact of spinal construct instability on the quality of life have also increased. In our study, 19 of the 24 patients in the F group (approximately 80%) required reoperation, highlighting the significant clinical burden of mechanical failure. Additionally, the time to symptomatic recurrence differed between the groups, with issues arising at an average of 6 months in the non-F group and 9.6 months in the F group. Complications related to spinal construct stability occurred within the expected survival period of these patients, making this an essential consideration for surgical planning.

The logistic regression analysis revealed that several factors were associated with implant failure after fixation-only surgery. PreopRT showed a trend toward significance (*p* = 0.077), with the placement of screws in areas of tumor involvement correlating with higher failure rates. However, factors such as the number of fixation levels and extent of laminectomy were not significantly associated with failure. Importantly, the multivariate analysis identified lower HU values at the LIV as the only independent predictor of failure. The ROC analysis determined a cutoff value of 127.27, below which the risk of implant-related failure due to weakened bone at the LIV was significantly increased. This finding was further confirmed by Kaplan–Meier survival analysis, where a significant difference (*p* = 0.0057) in survival rates between the groups with HU values above and below the cutoff was observed.

CT HU measurements are well established as predictive tools for implant failure in various spine surgeries [15,16], particularly in long-level deformity correction procedures [25,26,27]. In such surgeries, low HU values have been correlated with an increased risk of hardware-related complications, emphasizing the importance of bone quality assessment in surgical planning. Our study extends this knowledge to patients with metastatic spinal tumors, demonstrating that HU measurements at the LIV are critical in predicting the likelihood of implant failure. The identified cutoff value of 127.27 HUs provides a practical and clinically relevant threshold for assessing risk, further supporting the utility of CT HUs as a non-invasive predictor of surgical outcomes. Moreover, the predictive value of HU measurements for implant stability could have implications beyond spine surgery. For instance, in major joint replacement surgeries or other orthopedic procedures where bone quality significantly affects implant longevity, HU assessments could similarly aid in preoperative planning and risk stratification, potentially guiding the choice of stabilization techniques or implant materials [28,29].

One of the more intriguing findings of this study is the greater predictive power of LIV HU values than that of the UIV. Although both levels are crucial for maintaining spinal stability, the LIV may be more susceptible to mechanical failure than the UIV because of its position as the lower anchor point in the construct, where stress and load are maximized [5,30]. This finding aligns with those of studies in other contexts such as deformity correction, where the LIV plays a pivotal role in the overall integrity of the spinal construct [1,3,30]. For instance, in a study on degenerative lumbar scoliosis, Yuan et al. reported a loosening rate of 45.4% at the LIV compared to 17.7% at the UIV, suggesting that the primary cause of loosening was a weakened BMD at the LIV [31]. This observation is consistent with our findings, in which both representative cases (Figure 4 and Figure 5) demonstrated lower HU values at the LIV than at the UIV, leading to screw loosening at the LIV. Although anatomical factors such as screw trajectory and insertion angle may also contribute to loosening, within the heterogeneity of metastatic spinal tumors, the HU value at the LIV was the most objective predictor of loosening. Previous studies on degenerative conditions have similarly highlighted the significance of HUs in screw trajectory and its impact on outcomes [15,16]. Therefore, ensuring adequate bone quality at the LIV is essential for the long-term success of spinal instrumentation, particularly in patients with compromised bone quality.

Considering the critical role of LIV HU values identified in this study, alternative strategies for patients with HU values below the identified threshold of 127.27 need to be determined. This study provides a reference to guide clinicians and surgeons in their decision-making processes. For patients expected to have long-term survival, and thus a prolonged need for stable spinal instrumentation [32], surgical techniques such as the use of cement-augmented screws, thicker screws, or longer screws extending into the anterior VB may be considered [10,33,34]. Cement-augmented pedicle screws, which improve pull-out strength and reduce the risk of fixation failure in patients with osteoporosis or spinal metastases, could be particularly beneficial [34,35]. Unfortunately, in our study, only an average of 0.2% of cemented screws per patient were used in the F group, highlighting a potential area for improvement in surgical techniques. Additionally, the use of fenestrated pedicle screws with cementation further reduces the risk of screw loosening, which was a significant concern in our study [35]. While cement augmentation provides improved purchase power and stability, it is not without risks [35,36]. Cement leakage into the foramen can lead to root symptoms by compressing nearby nerves, and the inadvertent entry of cement into the venous system poses a risk of venous thrombus formation, which could result in serious complications [21,35,37]. Therefore, a careful technique and thorough intraoperative monitoring are essential when using cement-augmented screws to mitigate these risks.

These strategies can mitigate the risk of mechanical failure in high-risk populations. Future research should focus on evaluating the effectiveness of these techniques in improving outcomes for patients with low HU values at the LIV, as well as exploring the integration of other advanced imaging modalities or bone augmentation techniques [16,33,34,35]. Currently, advanced imaging techniques being developed and applied in other orthopedic fields include quantitative computed tomography (Q-CT) with phantom calibration, which allows for standardized, quantitative bone density assessments, and an AI-based volumetric analysis that evaluates bone structure in three dimensions [38,39]. These methods may provide a more detailed and precise assessment of bone quality, beyond traditional HU measurements, and could significantly enhance predictive accuracy for implant stability in patients with a compromised bone integrity.

The strength of this study lies in its large cohort size of 220 patients, including a focused analysis of 24 patients in the F group, representing approximately 10% of the cohort. The use of PSM to create a 1:3 ratio between the F and non-F groups minimized bias and strengthened the validity of the findings. This methodological rigor suggests that the results are reliable and applicable to similar patient populations. However, this study has some limitations. First, the retrospective design inherently carries the risk of selection bias, and the relatively small sample size of the F group may have limited the generalizability of the findings. Given the initial disparity in sample sizes between the F group and non-F group, we implemented propensity score matching (PSM) to create balanced groups for meaningful comparisons. Despite this approach, the limited size of the failure group remains a constraint. Although the analysis within the F group was limited by its size, gathering this number of failure cases at a single institution is a significant achievement. Second, the cohort included patients with metastatic spinal tumors across different spinal regions (cervical, thoracic, and lumbar), introducing a degree of heterogeneity that could influence the outcomes. The biomechanical and anatomical differences between these regions may have affected the risk of mechanical failure, which was not fully accounted for in this study. Additionally, while this study focused on key factors such as HU values and mechanical failure, other potential contributors, such as the number of tumor-affected segments, the duration of internal fixation, and the use of postoperative external fixation, may also influence fixation stability. Furthermore, our post hoc power analysis indicated a statistical power of approximately 72% with the current sample size, which is below the commonly recommended threshold of 80%. Future studies with larger, multicenter cohorts could provide additional insights by analyzing different spinal regions separately, to confirm whether these findings hold consistently across the cervical, thoracic, and lumbar segments. Finally, although HU values provide a valuable assessment of bone quality, they do not encompass all aspects of bone health, such as microarchitectural integrity, which may also influence implant stability.

## 5. Conclusions

This study suggests that preoperative bone quality assessment using CT HU measurements at the LIV may help in predicting the risk of mechanical failure following spinal instrumentation without fusion in patients with metastatic spinal tumors. While the identified HU cutoff value of 127.27 could serve as a useful tool for risk stratification, it should be considered as part of a comprehensive assessment of patient-specific risk factors rather than a standalone predictor. Personalized surgical approaches, particularly for patients with compromised bone quality, may benefit from incorporating HU measurements into preoperative planning. Future research is encouraged to validate these findings in larger prospective cohorts and to explore alternative surgical strategies for high-risk patients.

## Figures and Tables

**Table 1:** Demographic data of the mechanical failure and non-mechanical failure groups.

	Non-F Group	F Group	*p*
	(*n* = 72)	(*n* = 24)	
Age	58.4 ± 12.9	57.8 ± 13.1	0.856
Sex			1
Male	44 (61.1%)	14 (58.3%)	
Female	28 (38.9%)	10 (41.7%)	
Height	164.8 ± 9.5	166.6 ± 8.6	0.404
Weight	61.9 ± 10.3	64.7 ± 11.1	0.275
BMI	23.9 ± 6.5	23.3 ± 3.9	0.630
BMD	−1.9 ± 1.5	−2.1 ± 1.9	0.721
Follow-up period (months)	15.4 ± 15.8	21.6 ± 18.8	0.121
Involved vertebral bodies			0.731
1	27 (36.1%)	8 (33.3%)	
2	9 (12.5%)	5 (20.8%)	
≥3	36 (50.0%)	11 (45.8%)	
Pathologic fracture	53 (73.6%)	19 (79.2%)	0.785
Number of other bone lesions	1.1 ± 1.9	1.5 ± 3.5	0.551
Major organ metastasis	44 (61.1%)	17 (70.9%)	0.529
Mode of surgery			0.701
Fixation only	5 (6.9%)	1 (4.2%)	
Decompression and fixation	46 (63.9%)	14 (58.3%)	
Corpectomy and fixation	21 (29.2%)	9 (37.5%)	
PreopRT	24 (33.3%)	13 (54.2%)	0.115
PostopRT	50 (69.4%)	18 (75.0%)	0.795

BMI, body mass index; BMD, bone mineral density; RT, radiotherapy.

**Table 2:** Demographic and clinical summary of instrumentation failures in 24 patients.

Case No.	Sex/Age	Tumor Origin	Location	Mode of Surgery	No. of Laminectomy	Fixation Levels	Screws in Tumor	Failure Type	Time for Failure (Months)	Reoperation
1	M/50	Kidney	T5	Decompression and fixation	1	4	0	Failure c tumor recur	12	Yes
2	F/36	Breast	T12–L1	Fixation only	0	6	12	Failure	6	Yes
3	F/59	Lung	T12	Decompression and fixation	2	5	6	Failure c tumor recur	27	Yes
4	F/48	Kidney	T4	Corpectomy and fixation	1	4	0	Failure c tumor recur	17	Yes
5	M/57	Liver	C2	Decompression and fixation	1	2	0	Failure	1	Yes
6	M/71	Chondrosarcoma	T11	Corpectomy and fixation	2	4	1	Failure c tumor recur	1	Yes
7	M/72	Bladder	L3	Corpectomy and fixation	3	2.5	3	Failure c fracture	1	No
8	F/71	Kidney	T6	Corpectomy and fixation	1	4	0	Failure	2	Yes
9	M/71	MUO	L3–4	Decompression and fixation	3	4	0	Failure c tumor recur	10	Yes
10	F/27	Chondrosarcoma	T9	Decompression and fixation	5	2.5	0	Failure c fracture	0.25	Yes
11	F/47	Breast	L1	Decompression and fixation	1	2.5	3	Failure c tumor recur	2	No
12	F/56	Lung	L2,3	Decompression and fixation	3	3	1	Failure c fracture	2	Yes
13	M/75	Lung	L4	Decompression and fixation	2	2.5	1	Failure	3	No
14	F/60	Lung	L2–3	Decompression and fixation	2	5	5	Failure c tumor recur	14	Yes
15	F/72	Lung	T11–L1	Corpectomy and fixation	4	6	2	Failure	6	No
16	M/69	HCC	T11	Corpectomy and fixation	0	2	0	Failure c tumor recur	5	Yes
17	M/49	Lung	T8–9	Decompression and fixation	3	5	2	Failure c fracture	3	Yes
18	M/70	Liver	T10	Corpectomy and fixation	2	4	0	Failure c fracture	14	No
19	F/40	Breast	L4	Decompression and fixation	2	6	4	Failure	6	Yes
20	M/44	Thymus	T9	Corpectomy and fixation	3	5	0	Failure c fracture	18	Yes
21	M/63	Kidney	L4	Decompression and fixation	1	4	0	Rod breakage	14	Yes
22	M/70	Lung	L5	Decompression and fixation	1	4.5	3	Failure	5	Yes
23	M/56	Prostate	L3	Decompression and fixation	1	6	4	Failure	3	Yes
24	M/55	Lung	L1	Corpectomy and fixation	1	4	0	Rod breakage	26	Yes

M, male; F, female; MUO, metastasis of unknown origin; HCC, hepatocellular carcinoma; c, with.

**Table 3:** Characteristics of the mechanical failure and non-mechanical failure groups.

	Non-F Group	F Group	*p*
	(*n* = 72)	(*n* = 24)	
Bilsky grade			0.312
0	2 (2.8%)	3 (12.5%)	
1	8 (11.1%)	3 (12.5%)	
2	20 (27.8%)	6 (25.0%)	
3	42 (58.3%)	12 (50.0%)	
SINS	10.3 ± 3.5	11.4 ± 3.0	0.208
Location			0.21
Semi-rigid	29 (40.3%)	7 (29.2%)	
Mobile spine	12 (16.7%)	8 (33.3%)	
Junctional	31 (43.1%)	9 (37.5%)	
Pain			0.699
Pain-free	7 (9.7%)	1 (4.3%)	
Occasional but not mechanical	16 (22.2%)	6 (26.1%)	
Yes	49 (68.1%)	16 (69.6%)	
Bone lesion			0.813
Blastic	10 (13.9%)	2 (8.3%)	
Mixed	7 (9.7%)	3 (12.5%)	
Lytic	55 (76.0%)	19 (79.2%)	
Alignment			0.627
Normal alignment	42 (58.3%)	13 (54.2%)	
De novo deformity	28 (38.9%)	11 (45.8%)	
Subluxation/translation	2 (2.8%)	0 (0.0%)	
VB collapse			0.652
None	11 (15.3%)	3 (12.5%)	
No collapse with >50% body involved	14 (19.4%)	4 (16.7%)	
<50% collapse	27 (37.5%)	7 (29.2%)	
>50% collapse	20 (27.8%)	10 (41.7%)	
Posterolateral involvement			0.82
None	8 (11.1%)	2 (8.3%)	
Unilateral	21 (29.2%)	9 (37.5%)	
Bilateral	43 (58.3%)	13 (54.2%)	
UIV HUs	191.1 ± 79.2	161.0 ± 58.5	0.115
LIV HUs	178.9 ± 84.5	142.2 ± 62.2	0.042 *

SINS, spinal instability neoplastic score; VB, vertebral body; UIV, upper instrumented vertebra; LIV, lower instrumented vertebra; HU, Hounsfield unit. * *p* < 0.05.

**Table 4:** Surgical characteristics of the mechanical failure and non-mechanical failure groups.

	Non-F Group	F Group	*p*
	(*n* = 72)	(*n* = 24)	
Laminectomy levels	1.7 ± 1.2	1.9 ± 1.2	0.629
Fixation levels	3.7 ± 1.3	4.1 ± 1.3	0.279
Screws in tumor	1.0 ± 2.0	2.0 ± 2.8	0.139
No. of rods	2.0 ± 0.3	1.9 ± 0.2	0.503
No. of cemented screws	0.05 ± 0.4	0.2 ± 1.0	0.251
Symptomatic local recurrence	20 (27.8%)	12 (50.0%)	0.08
Time for symptomatic local recurrence	6.0 ± 8.4	9.6 ± 8.6	0.254
Reoperation	24 (33.3%)	19 (79.2%)	<0.001 *

No., number; * *p* < 0.05.

**Table 5:** Logistic regression analysis for factors related with postoperative implant failure.

	Estimate	Std. Error	z Value	Pr (>|z|)	OR	lcl	ucl
Age	0.001	0.0178	0.06	0.956	1	0.97	1.04
BMI	−0.0178	0.0463	−0.39	0.7	0.98	0.88	1.06
PreopRT	0.8408	0.4753	1.77	0.077	2.32	0.92	5.99
Bilsky grade	−0.3424	0.2543	−1.35	0.178	0.71	0.43	1.18
SINS	0.0906	0.0759	1.19	0.233	1.09	0.95	1.28
Mode of surgery	0.3228	0.4231	0.76	0.445	1.38	0.6	3.21
Laminectomy levels	0.1114	0.1921	0.58	0.562	1.12	0.76	1.62
Fixation levels	0.1943	0.1765	1.1	0.271	1.21	0.86	1.73
No. of cemented screws	0.3574	0.3414	1.05	0.295	1.43	0.7	3.37
No. of rods	−0.5444	0.8247	−0.66	0.509	0.58	0.09	3.42
Screws in tumor	0.1778	0.0976	1.82	0.068	1.19	0.99	1.46
UIV HUs	−0.0059	0.0038	−1.54	0.124	0.99	0.99	1
LIV HUs	−0.0051	0.0036	−1.43	0.153	0.99	0.99	1
(Intercept)	1.3423	0.9957	1.35	0.178	3.83	0.58	30.49
Bilsky grade	−0.4757	0.2865	−1.66	0.097	0.62	0.35	1.09
LIV HUs	−0.01	0.0048	−2.08	0.037 *	0.99	0.98	1

Residual deviance/df = 80.3/78 = 1.03, pseudo-R^2^ = 0.421 (Nagelkerke). BMI, body mass index; PreopRT, preoperative radiotherapy; SINS, spinal instability neoplastic score; No., number; UIV, upper instrumented vertebra; LIV, lower instrumented vertebra; HU, Hounsfield unit; OR, odds ratio; lcl, lower confidence limit; ucl, upper confidence limit. * *p* < 0.05.

## Data Availability

Data are available upon reasonable request.
